# Bacteriological and Phytochemical Assessment of *Ficus asperifolia* Linn. Infusion

**DOI:** 10.1155/2020/9762639

**Published:** 2020-05-13

**Authors:** Kolapo Ayoola Fasina, Titilayo O. Adesetan, Faithfulness Oseghale, Haneefat O. Egberongbe, O. O. Aghughu, Fred A. Akpobome

**Affiliations:** ^1^Microbiology Department, Faculty of Science, Olabisi Onabanjo University, Ago Iwoye, Ogun State, Nigeria; ^2^Rubber Research Institute of Nigeria, Benin City, Edo State, Nigeria; ^3^Agronomy Department, Rubber Research Institute of Nigeria, Benin City, Edo State, Nigeria

## Abstract

*Ficus asperifolia* Linn. known as “Eepin” in Yoruba language, or sand paper tree, is a monoecious fig tree whose leaves, bark, seeds, and roots have been used locally in treating many infectious and noninfectious diseases. The study is aimed at investigating the bacteriological and phytochemical potential of *Ficus asperifolia* Linn. The roots of the plant were harvested and washed, and phytochemical analysis was carried out using standard analytical techniques. Infusion was aseptically prepared, and incubation for 24 hours and microbiological analysis were carried out using the pour plate method on Plate Count Agar (PCA) and Nutrient Agar (NA). Microorganisms were subcultured and identified using morphological and biochemical tests according to “Bergey's Manual of Determinative Bacteriology.” Phytochemical analysis of the fresh and dry roots revealed the presence of alkaloids, cardenolides, and saponins, while anthraquinones and tannins were absent. Total heterotrophic bacteria count on PCA was 5.6 × 10^5^ CFU/ml, while on NA, it was 2.3 × 10^5^ CFU/ml, and four classes of bacteria were isolated including *Klebsiella* sp., *Escherichia coli*, *Proteus* sp., and *Bacillus* sp. Although the presence of medicinal phytochemicals in *F. asperifolia* Linn. indicates strong potentials for its use in infusions, the presence of potential pathogens found in the infusions makes it unsafe for consumption.

## 1. Introduction

The genus *Ficus* Linn. is made up of close to 1000 species throughout tropical and warm temperate regions, showing diversity in South East Asia, Malaysia, and tropical South America, with 42 species in Australia [1]. Berg [2] reported about 105 species in the African floristic region with some 5 dozen in West Africa, and at least 44 species in Nigeria. Thought to be the sweetest fruit, figs are also one of the oldest fruits recognized by man. Its sweet delicious flesh, long used as a sweetener before the advent of refined sugars, enhances both savoury dishes and desserts [3].

Plant roots release a wide variety of materials to their surrounding soil including various alcohols, ethylene, sugars, amino acids and organic acids, vitamins, nucleotides, polysaccharides, and enzymes. These materials create a unique environment for the soil microorganisms [4].

The water used in the preparation of herbal concoctions and poultices is a very important factor, which could be a source of contamination with enteric organisms. Annan and Houghton [5] reported that *Ficus asperifolia* Linn. lacked antimicrobial properties.

There appears to be clinical, scientific, and pharmacological basis for the use of herbal preparations. Ajose [6] stressed that “Nigeria therefore needs to provide effective coordination of the practice of herbal medicine to ensure safety, standardization and preservation of the flora” to awaken us to the dangers associated with wrong prescription and administration of herbal remedies today.

The aim of this study was to evaluate the phytochemical composition of *Ficus asperifolia* Linn., as well as evaluate the bacteriological content of the cold infusion.

## 2. Materials and Methods

### 2.1. Study Location, Sample Collection, and Identification

The roots of the plant *Ficus asperifolia* Linn. were collected from the Forestry College of Technology, Idi-Ishin, Ibadan, Oyo State, Nigeria, and taken to a taxonomist at the Botany and Microbiology Department, University of Ibadan, Ibadan, Oyo State, Nigeria, for adequate identification.

### 2.2. Sample Preparation

The roots of *F. asperifolia* were transported to the laboratory where analysis was carried out on it. Fifty grams (50 g) of the root sample was put into an airtight plastic bottle, and 400 ml of refined (eva) water was then added to the root in order to prepare the infusion. It was then closed with the cover and left for 3 days before bacterial isolation was carried out.

### 2.3. Isolation of Bacteria

The serial dilution was carried out as described by Prescotte et al. [4]. The bottle containing the infusion was shaken vigorously and 1 ml was pipetted aseptically into 9 ml of sterile water in test tubes to achieve 10^−1^ dilution of the sample. The test tubes were plugged with sterile cotton wools and foil paper to avoid contamination. Additional 1 ml aliquots were transferred into test tubes with 9 ml of sterile distilled water to prepare the fourth dilution. Then, 1 ml of the fourth dilution was pipetted onto sterile petri dishes, and molten agar (Plate Count Agar and Nutrient Agar) of about 45°C was poured into the plate for bacteria growth. Seeded plates were incubated at 37°C for 24 hours and observed for growth. Plates containing between 30 and 300 colonies were counted and recorded.

Distinct colonies were subcultured on fresh plates using a wire loop and later transferred onto sterile slants and stored at 4°C.

### 2.4. Bacteria Characterization

Pure cultures of bacteria isolates were characterized using morphological and biochemical tests according to “[7].” The colonial shapes, elevations, edges, pigmentations, and consistencies were observed and recorded. Biochemical tests that were carried out include Gram staining, catalase test, citrate utilization test, and sugar fermentation tests (glucose, fructose, sucrose, D-galactose, maltose, and lactose). Inferences were made and bacteria identities were suggested.

### 2.5. Phytochemical Screening Tests

Portions of fresh and dry powders of the root of *F. asperifolia* Linn. were prepared and screened for the presence of phytochemicals including alkaloids, cardenolides, saponins, tannins, anthraquinones. Screening was carried out at the Phytochemistry Laboratory, Pharmacognosy Unit, University of Ibadan, Ibadan, Oyo State, Nigeria.

### 2.6. Alkaloids

An alkaloid test was done according to the method of Harborne [8]. Powdered samples (1 g each) were added to 10 ml of 10% HCl for extraction by shaking vigorously and then filtering. Dragendoff's reagent (1 ml each) was added into separate test tubes of the filtrate. Observations for brown ring formation were made and recorded. Mayers' and Wagner's reagents were also used and observations recorded.

### 2.7. Cardenolide Test

Powdered samples (1 g) were added to 10 ml of 80% alcohol and shaken vigorously. It was filtered and then lead acetate, water, and chloroform were added and allowed to evaporate to dryness. Ferric chloride and concentrated sulphuric acid were then added according to the method of Trease and Evans [9]. Observations for brown ring formation were made and recorded.

### 2.8. Anthraquinones

Powdered samples (1 g) were added into 10 ml of 10% HCl for extraction and shaken vigorously, and afterwards, it was filtered. Chloroform (5 ml) was then added to the filtrate, and the chloroform was carefully decanted from the filtrate. Three millilitres of 10% ammonia solution was added, and observations were made afterwards and recorded.

### 2.9. Tannins

A test for tannins was carried out as described by Adeyemi et al. (2005) by adding 10 ml of boiling water to 1 g of each sample. The samples were then filtered using filter paper, after which 1 ml of ferric chloride was added to each filtered sample. Observation for the formation of precipitates was made and recorded.

### 2.10. Saponins

The extract was subjected to a frothing test for the identification of saponins. One gram of each of the powdered samples was put into 10 ml of distilled water, shaken vigorously to dissolve samples. Frothing reaction was observed and recorded.

## 3. Results

### 3.1. Total Viable Bacteria Counts

Total viable bacteria counts from the *F. asperifolia* Linn. infusion was 5.6 × 10^5^ CFU/g on PCA, while the count on NA was 2.3 × 10^5^ CFU/g.

Four groups of bacteria were isolated from the *Ficus asperifolia* Linn. infusion including *Klebsiella* sp., *Escherichia coli*, *Proteus* sp., and *Bacillus* sp. (Table 1).

The phytochemical analysis exhibited the presence of alkaloids in the fresh roots of the plant (Table 2). Anthraquinones, cardenolides, and saponins were found to be present, while anthraquinones and tannins were absent. While the presence of alkaloids was confirmed to be present with Dragendoff's reagent and Wagner's reagents, Meyers' reagent did not support its presence. Also, the presence of cardenolides was confirmed when Keller-Killiani's procedure was used, whereas it wasn't found with Kedde's procedure.

Similar to the results found in fresh roots, the phytochemical analysis exhibited the presence of alkaloids in the fresh roots of the plant (Table 3). Anthraquinones, cardenolides, and saponins were found to be present, while anthraquinones and tannins were absent. While the presence of alkaloids was confirmed to be present with Dragendoff's reagent and Wagner's reagents, Meyers' reagent did not support its presence. Also, the presence of cardenolides was confirmed when Keller-Killiani's procedure was used, whereas it wasn't found with Kedde's procedure.

## 4. Discussion

This study elucidated the presence of diverse kinds of bacteria in the infusion made from *Ficus asperifolia*, some of which are of public health importance. These include *Escherichia coli*, *Klebsiella* sp., *Bacillus* sp., and *Proteus* sp. Coliforms such as *Escherichia coli* are facultative anaerobes with an optimum growth at 37°C, and they serve as indicators of fecal contamination from pathogenic organisms [10]. They usually cause rapid spoilage [11], and their consumption can lead to diarrhea [12]. *E. coli* are gram negative rods that are commonly found as part of the flora in the gastrointestinal tract of humans and animals.

*E. coli* is now recognized as an important foodborne disease organism. Enteropathogenic, enteroinvasive, and enterotoxigenic types can cause diarrhea.

The presence of *Klebsiella* sp. also indicates the presence of fecal contamination [13]. *Klebsiella* are large motile bacteria that possess a luxurious capsule and lactose fermenters. *K. pneumoniae* and *K. oxytoca* cause a necrotizing lobar pneumonia in individuals compromised by alcoholism, diabetes, or chronic obstructive pulmonary disease. *K. pneumoniae* also causes urinary tract infections and bacteremia, particularly in hospitalized patients [14]. *Proteus* sp., a peritrichous flagellated motile swarmer, was also isolated in this study. These organisms swarm on solid media, spreading rapidly in a thin film resulting from periodic cycles of migration. They occur in the intestines of humans and a wide variety of animals, in polluted waters, and in soil.

Another important horizon from which to interpret the roles of these bacteria would be to look at them as plant-growth-promoting bacteria, where they might be secreting substances such as indole acetic acid or naphthalene acetic acid to support root development. They may also act as biocontrol agents to protect the plant from pathogens.

It is not yet known what the source of contamination could have been. However, knowing that *E. coli* and *Klebsiella* spp. are coliforms could point to the water used in preparing the infusion as the source of contamination, since it was not sterilized or cooked before infusing the plant roots. The presence of *Proteus* sp. in the roots might also indicate that the contamination comes from the roots themselves, since they were harvested from the soil and washed thoroughly with treated sachet water before infusing. Most infusions made are usually prepared without proper care about the sterility of the surface of the plant parts as well as the water being used for its preparation. The water used in the preparation of local infusions should, at the least, adhere to the World Health Organisation's standards of potability to prevent water-borne pathogen inoculation of the infusion, thereby preventing contamination, ultimately making it fit for human consumption.

The phytochemical assay on the roots and stem of *Ficus asperifolia* showed the presence of alkaloids, anthraquinones, saponins, and tannins. This outcome agrees with the work done by Preeti et al. [15] who found plant extracts from *Ficus elastica* to be rich in antioxidant activity as well as phytochemicals such as flavonoids, phenolics, and tannins. Eleazu et al. [16] also worked on five plant extracts (bitter kola, neem, tetrapluera, pawpaw, and ginger) and found them to contain various phytochemicals of potential antimicrobial activity including saponins, tannins, alkaloids, flavonoids, and HCN. Akinmoladun et al. [17] mentioned that flavonoids have antioxidant potentials, showing therapeutic potentials against potential pathogens.

Saponins have been connected with precipitating and coagulating red blood cells (hemolysis), cholesterol binding activity, and characteristic formation of foams in aqueous solutions [18]. They also exhibit a bitter taste [17]. The presence of saponins in *Ficus asperifolia* suggests that caution should be taken when administering the infusion, so as to avoid any hemolysis of the patient's blood. Other studies on plant extracts with moderate amounts of saponins have shown no toxicological effect on hosts.

Alkaloids are highly efficient basic natural products that are present in many plants, and they have been used in many analgesic, antiplasmodic, and antibacterial medications [18]. The presence of alkaloids in the plant extract indicates that they may confer protection against allergies, microbes, ulcers, viruses, platelet aggregation, and tumor [19].

The presence of flavonoids in *Ficus asperifolia* extracts, which are water-soluble antioxidants with anticancer activity [19], also portends that its infusion will have therapeutic properties.

## 5. Conclusion and Recommendation

*Ficus asperifolia* Linn. has been reportedly used in herbal remedies for treating many venereal diseases, oedema, and nasopharyngeal infections. The infusion of the roots has also been used in treating wound infections as well as cough. This study has shown that the plant possesses some vital phytochemicals which suggests its use in herbal remedies. However, the infusion could be a reservoir of plant-growth-promoting bacteria, which could be biocontrol agents for protecting the plants against pathogens. These bacteria may/may not be potential human pathogens when adequate hygienic conditions are not observed during its preparation. It may be healthier to prepare decoctions instead of cold infusions.

The authors therefore recommend that further studies should be carried out on decoctions (warm water infusion) of *Ficus asperifolia* Linn., to observe the effect of higher temperatures on plant microbes, and yet preserve the efficacy of the phytochemicals in the plant extracts.

## Figures and Tables

**Table 1:** Colonial morphology and biochemical tests of microbial isolates.

Characteristics	PCA		NA	
	N1	N2	N3	N4
Shape	Circular	Irregular	Irregular	Circular
Elevation	Convex	Unbonate	Flat	Flat
Edge	Entire	Entire	Lobulated	Entire
Consistency	Moist	Dry	Moist	Dry
Opacity	Opaque	Opaque	Translucent	Opaque
Spread	None	None	Swarming	None
Pigmentation	Cream	White	Creamish white	Creamish white
Grams	-ve	-ve	-ve	+ve
Shape	Rod	Rod	Rod	Rod
Catalase	+ve	-ve	-ve	+ve
Citrate	+ve	-ve	-ve	+ve
Sucrose	+ve	+ve	+ve	+ve
Maltose	-ve	+ve	+ve	+ve
Fructose	+ve	+ve	+ve	+ve
Glucose	+ve	+ve	+ve	+ve
D(+) galactose	+ve	+ve	-ve	+ve
Lactose	-ve	+ve	-ve	+ve
Probable organism	*Klebsiella* sp.	*Escherichia coli*	*Proteus* sp.	*Bacillus* sp.

**Table 2:** Phytochemical analysis of the fresh roots of *Ficus asperifolia*.

Test	Observation	Inference
Alkaloid test		
(i) Dragendoff's reagent	Brown precipitate formed	Alkaloids present
(ii) Meyer's reagent	No precipitate formed	Alkaloids absent
(iii) Wagner's reagent	Brown precipitate formed	Alkaloids present
Cardenolide test		
(i) Keller-Killiani	Brown ring formed	Cardenolides present
(ii) Kedde	No brown ring	Cardenolides absent
Anthraquinone test	No mixture of chloroform and ammonia	Anthraquinones absent
Saponin test (frothing)	Soapy froth observed	Saponins present
Tannin test (ferric chloride)	No precipitate	Tannins absent

**Table 3:** Phytochemical analysis of the fresh roots of *Ficus asperifolia* Linn.

Test	Observation	Inference
Alkaloid test		
(i) Dragendoff's reagent	Brown precipitate formed	Alkaloids present
(ii) Meyer's reagent	No precipitate formed	Alkaloids absent
(iii) Wagner's reagent	Brown precipitate formed	Alkaloids present
Cardenolide test		
(i) Keller-Killiani	Brown ring formed	Cardenolides present
(ii) Kedde	No brown ring	Cardenolides absent
Anthraquinone test	No mixture of chloroform and ammonia	Anthraquinones absent
Saponin test (frothing)	Soapy froth observed	Saponins present
Tannin test (ferric chloride)	No precipitate	Tannins absent

## Data Availability

The data used to support the findings of this study may be released upon application to the corresponding author.
